# Influence of the long-term use of oral hygiene products containing stannous ions on the salivary microbiome – a randomized controlled trial

**DOI:** 10.1038/s41598-020-66412-z

**Published:** 2020-06-12

**Authors:** A. C. Anderson, A. Al-Ahmad, N. Schlueter, C. Frese, E. Hellwig, N. Binder

**Affiliations:** 1 Department of Operative Dentistry and Periodontology, Medical Center-University of Freiburg, Faculty of Medicine, University of Freiburg, Freiburg, Germany; 2 Division for Cariology, Department of Operative Dentistry and Periodontology, Medical Center- University of Freiburg, Faculty of Medicine, University of Freiburg, Freiburg, Germany; 3 Department of Conservative Dentistry, Clinic for Oral, Dental and Maxillofacial Diseases, University Hospital Heidelberg, Heidelberg, Germany; 4 Institute for Prevention and Cancer Epidemiology, Faculty of Medicine and Medical Center, University of Freiburg, Freiburg, Germany; 5 Institute of Digitalization in Medicine, Faculty of Medicine and Medical Center, University of Freiburg, Freiburg, Germany

**Keywords:** Microbiology, Microbial communities, Microbiome, Dental diseases

## Abstract

Oral hygiene products containing tin are suitable to prevent erosive tooth wear, yet effects on the oral microbiota are not known yet. Therefore, this study determined the salivary microbiome of 16 participants using products with stannous ions for three years (TG) compared with a control group (CG) to assess their influence on the microbiota. Participants were included in a randomized controlled clinical trial (RCT) with biannual visits. Illumina Miseq sequencing revealed as most abundant genera: *Streptococcus* (TG 14.3%; CG 13.0%), *Veillonella* (TG 11.3%; CG 10.9%), *Prevotella* (TG 7.0%; CG 9.8%), *Haemophilus* (TG 6.6%; CG 7.2%), *Porphyromonas* (TG 5.9%, CG 5.1%), *Leptotrichia* (TG 5.8%; CG 4.9%), *Actinomyces* (TG 4.0%; CG 4.6%) and *Neisseria* (TG 5.4%; CG 4.2%). Beta-Diversity was not significantly different between groups at both time points, although significant differences between groups were found for certain taxa after three years. The genus *Prevotella* was found in higher abundance in CG whereas *Neisseria* and *Granulicatella*, health-associated taxa, were found more abundantly in TG. Salivary microbiota after three years reflected a composition associated with oral health, hence continual use as a preventive measure for dental erosion can be considered safe and benefitting oral health for patients with a high risk of erosion.

## Introduction

Erosive tooth wear, a loss of dental hard tissue due to the direct impact of acids occurs worldwide with a prevalence of 20 to 40%^1^. Diets high in acidic beverages and foods, gastro-esophageal reflux and eating disorders, e.g. bulimia, are among the factors that provoke this gradual loss of enamel and dentine^2^. The anti-erosive effect of stannous compounds has been studied in the last decades. Products containing stannous ions in combination with fluoride (F/Sn) have been found to exert marked anti-erosive effects, reducing tissue loss in acidic challenges in numerous studies^3–6^ and are considered standard^7^. The stannous compounds form a protective layer on the enamel surface and they can be incorporated into the enamel under erosive conditions protecting the underlying structures against erosive challenges^8,9^. This data emphasizes the erosion-preventive effect of these products. Oral hygiene products containing stannous ions also have an antibacterial effect and are therefore potentially beneficial concerning prevention of caries and periodontitis. To date many studies have analyzed the effect of stannous ions on selected microbial species or the general bacterial count, yet it has not been understood how these compounds influence the microbial community composition as a whole^10–13^. Furthermore, the long-term effect of these products on the oral microbiota *in vivo* has not been studied so far.

Salivary microbiota, similar to other oral niches, contain commensal microorganisms that -in health- comprise a physiological flora that is beneficial as long as homeostasis is maintained^14^. Environmental disturbances can increase the abundance of potentially pathogenic species leading to oral diseases, such as caries and periodontitis^15,16^. Therefore excessive eradication of the resident flora is not expedient, but it would be desirable to selectively influence the proportion of potentially pathogenic species^17^. In recent years, the salivary microbiome has been studied more extensively. The findings suggest a salivary core microbiome being present in the majority of individuals, accounting for a very large proportion of determined sequences and being fairly stable temporally^18–20^. The salivary microbiota resemble various oral sites, e.g. the tongue, the supragingival as well as subgingival plaque and are thought to be shaped by microorganisms deriving from these niches^21^. Certain taxa in saliva have been associated with specific oral diseases, e.g. periodontitis, early childhood caries or with oral health^21–23^. Therefore, besides investigating the clinical efficacy of oral hygiene products containing stannous ions, it is essential to investigate their impact on the microbial community. Hence, the aim of our study was to analyze the influence of the long-term use of a mouthrinse and toothpaste containing SnCl_2_/AmF/NaF over three years on the salivary microbiome of an intervention group compared to a control group using products containing AmF/NaF alone. To achieve this objective, we applied high-throughput DNA amplicon sequencing based on the 16S rDNA on the Illumina MiSeq platform.

## Results

### Participant data

Fifty-four volunteers were recruited, 41 male and 13 female (mean age 36.53 ± 9.49 years; range 20–60 years), 27 for each group. The overall dropout rate was 29.6% with 5 subjects resigning from the control, and 11 subjects from the test group. Table 1 gives the summarized demographic and clinical parameters; detailed data are given in Supplementary Table S1. Supplementary Fig. S1 shows the flow chart according to CONSORT.

**Table 1:** Demographic and clinical data of study participants (n = 38) at baseline and after three years (t = 0, t = 3). (Data modified from^33^).

Study group	BMI [kg/m^2^]*	Age [years]*	sex		Saliva pH*		Saliva flow rate [ml/min]*	
	t = 0	t = 0	Male	Female	t = 0	t = 3	t = 0	t = 3
TG	23.06 ±2.59	38.0 ±10.85	12 (75%)	4 (25%)	7.05 ±0.69	6.76 ±0.32	2.33 ±0.97	1.98 ±0.70
CG	23.01 ±2.35	38.7 ±9.50	19 (86%)	3 (13%)	6.83 ±1.19	6.72 ±0.36	2.15 ±0.90	1.87 ±0.82

*(mean ± SD).

### Composition of the salivary microbiota in TG and CG

**Figure 1:**
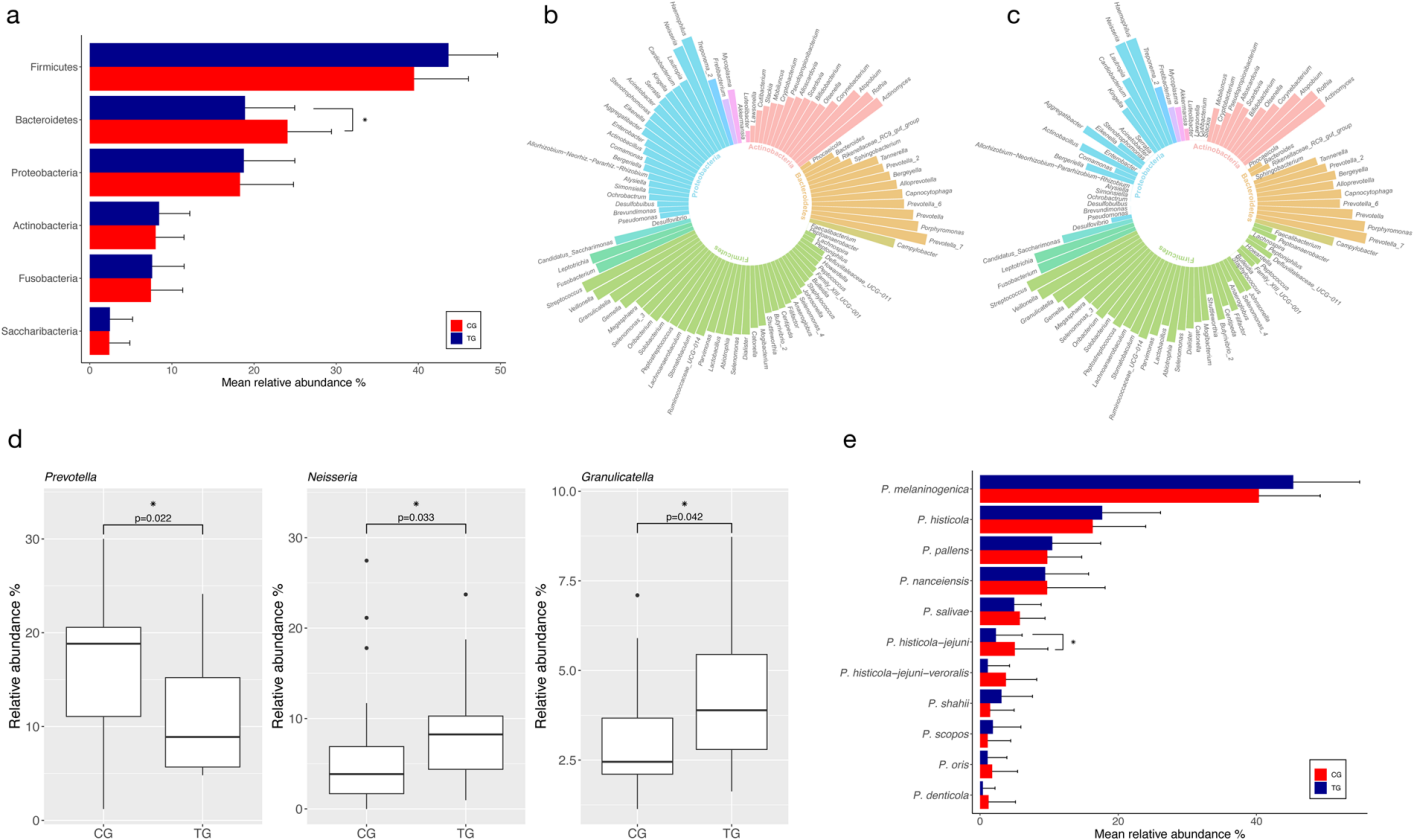
(**a**) Relative abundances of the top six phyla (>1% abundance) detected in saliva in TG (Treatment group, blue) and CG (Control group, red) (*p ≤ 0.05) (**b**) Relative abundance of the bacterial genera detected in saliva in CG, (**c**) Relative abundance of the bacterial genera detected in saliva in TG, (**d**) Relative abundances of the genera *Prevotella*, *Neisseria* and *Granulicatella* in CG and TG, (**e**) Relative abundances of the different *Prevotella* species (>1% abundance) in TG (blue) and CG (red), (*p ≤ 0.05).

Illumina Miseq sequencing of the 38 remaining saliva samples at T3 (three years) achieved an average of 120,649 raw reads per sample which resulted in an average 29,106 reads per sample after trimming, merging and quality filtering (Zymo Research Data). Taxonomic annotation based on Zymo Research Database (Zymoresearch, USA) using Uclust from Qiime (v.1.9.1, Caporaso *et al*. 2010) resulted in 285 different bacterial species belonging to 81 genera and 11 phyla. The most abundant phyla were Firmicutes (TG 43.7%; CG 39.5%), Bacteroidetes (TG 18.9%, CG 24.1%; significantly higher in CG; p = 0.045), Proteobacteria (TG 18.8%; CG 18.3%), Actinobacteria (TG 8.4%; CG 8.0%) and Fusobacteria (TG 7.6%; CG 7.4%) as depicted in Fig. 1a and Supplementary Fig. S2. For comparison, the DADA2 software pipeline based on Silva database v132 was used and resulted in 89 different bacterial genera belonging to 10 phyla. The most abundant phyla found in both groups were Firmicutes (TG 41.6%; CG 38.8%), Bacteroidetes (TG 20.4%; CG 24.3%), Proteobacteria (TG 14.0%; CG 13.7%), Actinobacteria (TG 9.6%; CG 9.65%) and Fusobacteria (TG 9.1%; CG 8.0%). The most abundant genera detected were *Streptococcus* (TG 14.3%; CG 13.0%), followed by *Veillonella* (TG 11.3%; CG 10.9%), *Prevotella* (TG 7.0%; CG 9.8%), *Haemophilus* (TG 6.6%; CG 7.2%), *Porphyromonas* (TG 5.9%, CG 5.1%), *Leptotrichia* (TG 5.8%; CG 4.9%), *Actinomyces* (TG 4.0%; CG 4.6%) and *Neisseria* (TG 5.4%; CG 4.2%). Figure 1b,c shows the abundances for CG and TG. The alpha diversity between TG and CG did not differ, but the species richness was significantly lower in TG, (TG 90 obs. sp., CG 106.8; p = 0.047; based on Zymo Research data; Supplementary Table S2) At T0 (baseline), the same most abundant phyla and genera were detected in both groups (depicted in Supplementary Figs. S3–S5). The full list of OTUs and ASVs and their relative abundances are given in Supplementary Table 4 and 5. In the following we will focus on the results based on the Zymo Research Database.

**Figure 2:**
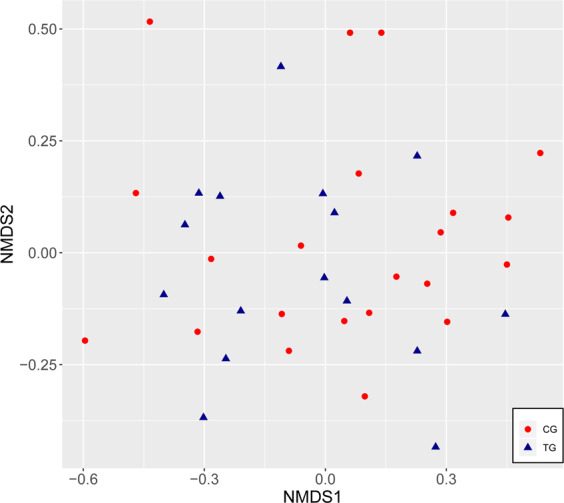
NMDS plot (non-metric multidimensional scaling) depicting the beta-diversity of the microbiota in saliva in CG and TG (Zymo Research Data).

In order to assess the differences between the salivary microbiome of the two groups, the beta-diversity was analyzed on the basis of the Bray-Curtis dissimilarity. The non-metric multidimensional scaling did not show any distinct clustering in either TG or CG at T0 nor at T3, and there were no significant differences in the calculated beta-diversity (permutational multivariate analysis of variance based on Bray-Curtis dissimilarity; p = 0.447 at T0; p = 0.302 at T3.) (Fig. 2). Consequently, the salivary bacterial community as a whole is not significantly affected by the long-term use of products containing SnCl2/AmF/NaF.

**Figure 3:**
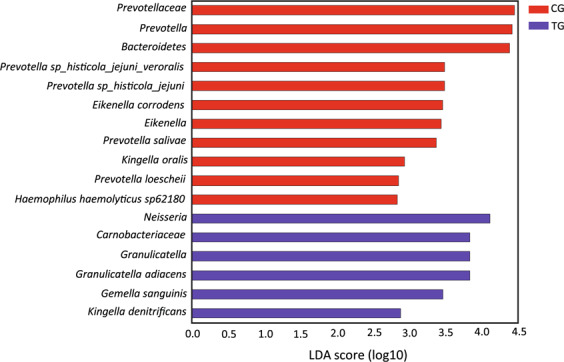
Bar graph of LEfSe analysis of bacterial taxa in saliva in CG (red) and TG (blue) showing LDA scores (Biomarkers sorted according to effect size).

**Figure 4:**
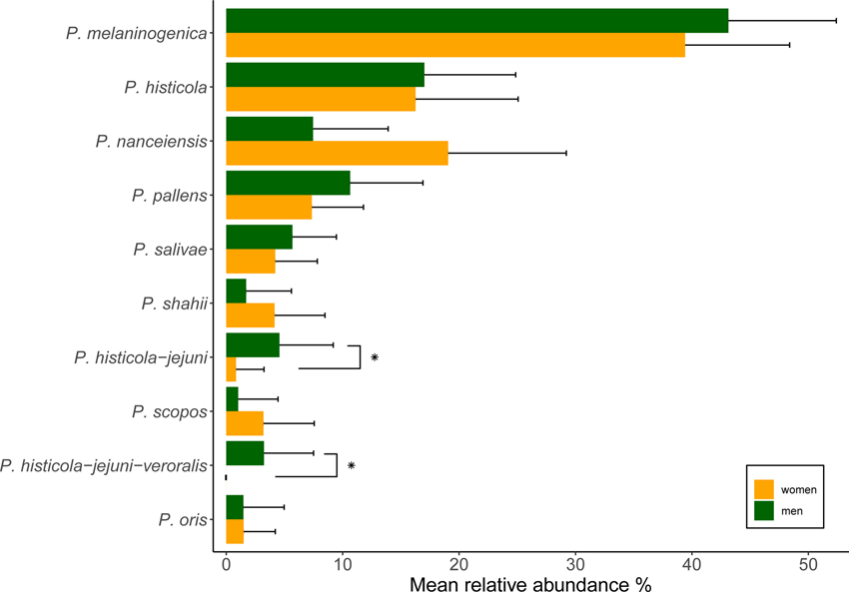
Relative abundances of the different *Prevotella* species (>1% abundance) in saliva of both groups related to the gender of the study participants (green = men, orange = women), (*p ≤ 0.05).

Even though there was no significant difference in beta-diversity, certain taxa showed a significantly different abundance in the two groups at T3, but not at T0. A LEfSe analysis revealed that *Prevotella* taxa, *P. histicola_jejuni_veroralis*, *P. salivae* and *P. loescheii* as well as *Eikenella* sp., *Kingella oralis* and *Haemophilus haemolyticus* were found in significantly higher abundance in CG. *Neisseria* sp., *Granulicatella* sp., *Gemella sanguinis* and *Kingella denitrificans* were found enriched in TG (Fig. 3). Separate Wilcoxon tests confirmed this, showing a significantly higher abundance of the genus *Prevotella* in CG (p = 0.022) and a significantly higher abundance of the genus *Neisseria* (p = 0.033) as well as *Granulicatella* (p = 0.042) in TG (Fig. 1d). The analysis of the sequences on the species level revealed that certain *Prevotella* species were more abundant in CG (e.g. *P. histicola_jejuni* p = 0.041; *P. histicola_jejuni_veroralis*, n.s.) (Fig. 1e; Supplementary Fig. S6). At baseline, these taxa did not show any significant differences (Supplementary Fig. S7). When the abundance of *Prevotella* species was related to the distribution of males and females in the two groups, significant gender-related differences were revealed (Fig. 4). Thus, 2 *Prevotella* taxa were significantly more abundant in males (e.g. *P. histicola_jejuni*, p = 0.035; *P. histicola_jejuni_veroralis* p = 0.015). The genera *Neissera* and *Granulicatella* did not show any gender-related differences in their abundance.

As far as the mock community used as positive control, the Illumina MiSeq sequencing detected all taxa included in the mock community. Contaminant reads were ≤5 reads per sequenced sample. The relative abundances of the included taxa were not equal, *Tannerella* sp. and the two *Streptococcus* species, which were not differentiated (*S. mitis* and *S. sanguinis*) were underrepresented and on the other hand *Porphyromonas* sp. and *Fusobacterium* s.p. were somewhat overrepresented. The results of the mock community sequencing are shown in Supplementary Table S3.

## Discussion

Oral hygiene products containing stannous ions are suitable for continuous use as preventive measures^24^, however, their long-term influence on the oral microbiota has not been studied yet. Therefore, this study aimed to analyze the salivary microbiome of individuals with elevated risk for dental erosion that used products containing SnCl_2_/AmF/NaF over a period of three years in comparison to a control group. Since the salivary microbiota contains bacteria shed from different oral sites its composition is thought to reflect the oral microbiota as a whole^21,25^. Therefore there is an increased interest in sampling saliva to determine oral health, and to our knowledge this is the first study that investigated the effect of a long-term use of such products on the salivary microbiota *in vivo*. The most noteworthy outcome was that no significant difference could be observed in the microbial composition in TG compared to CG at both time points, even though certain taxa showed significantly different abundances in the two groups at T3. Overall, the microbiome of TG after the three-year treatment period revealed a composition of microbial taxa that can be interpreted as health-associated.

Previous studies have proven the ability of stannous ions to reduce plaque and salivary bacterial count. Addy *et al*. tested their effect on the salivary bacterial count and on plaque regrowth and reported a higher reduction compared to the control^10^. Auschill *et al*. evaluated the influence on the biofilm thickness of plaque and the bacterial viability and reported significant reduction of both parameters compared to the control^26^. Other authors tested the effect *in vitro* against single species or artificial biofilms consisting of certain oral taxa, e.g. *Porphyromonas gingivalis*, *Fusobacterium nucleatum*, *Streptococcus mutans*, *S. sanguinis* and reported the efficacy of these compounds to reduce bacterial growth^11,13,27^. Cheng *et al*. found growth of *S. mutans* and *P. gingivalis* suppressed whereas *S. sanguinis* was enriched after five days. Shapiro *et al*. found differentially reduced growth for several species in a six-species biofilm, with *F. nucleatum* and *S. oralis* reduced the most^13^.

However, in contrast to our study, the focus of these authors was the short-term effect of these products to reduce the bacterial load until plaque regrowth occurred. They failed to take into account the microbial community as a whole *in vivo* reacting to outward influences as an ecological framework with specific interactions between the taxa present. Our study used comprehensive high-throughput sequencing revealing the influence of the long-term use of stannous chloride on the whole microbial community. The samples of both groups, TG and CG revealed a high abundance of the genera *Streptococcus*, *Veillonella*, *Prevotella*, *Granulicatella*, *Gemella*, *Haemophilus*, *Neisseria*, *Porphyromonas*, *Actinomyces* and *Rothia*, corresponding to the taxa found as salivary core microbiome in many recent studies^20,28,29^. Regarding the species diversity, both groups did not differ from each other at T3 which is indicative that the treatment groups’ microbiota represents a healthy status. Kumar *et al*. argue that similar levels of diversity are important, since any deviation from the controlled diversity in the oral ecosystem associated with a healthy status appears to result in a dysbiotic condition, eventually leading to disease^30^. On the other hand, the species richness was lower in TG, which is also indicative of a healthy status^20,31^.

As for differentially abundant taxa found in the two groups at T3, the genera *Prevotella*, *Neisseria* and *Granulicatella* stand out. The genus *Prevotella* was found in significantly higher abundances in CG (Fig. 1d). In part this could be attributed to the fact that the control group included more men and *P. histicola-jejuni* and *P. histicola-jejuni-veroralis* were significantly more abundant in males. Zaura *et al*., analyzing the salivary microbiome of 268 individuals also reported a higher abundance of *Prevotella* spp. in males, whereas females showed a higher abundance of certain *Streptococcus* species^29^. Yet, since the whole genus *Prevotella*, showed a significantly higher abundance in CG it can be assumed that stannous chloride might suppress the growth of many representatives of this taxon.

Interestingly, Yang *et al*., studying salivary microbiomes of caries-active and healthy individuals, found an association of *Prevotella* sp. (*P. histicola* among others) with caries-active individuals which was confirmed by Teng *et al*., who reported an increased abundance of various *Prevotella* species in the saliva of children with early childhood caries^22,23^. According to their assumption, this taxon could be indicative of a caries-active status. Belstrom *et al*., comparing saliva of healthy individuals with caries and periodontitis patients, showed that traditional periodontal pathogens and cariogenic species, were found significantly more often in the saliva from patients with the respective condition^21^. As for *Prevotella* taxa, *P. intermedia*, *P. baroniae* and *P. falsenii* were enriched in periodontitis patients, *P. denticola* in caries patients, yet *P. scopos* and *P. bergensis* were associated with oral health. Regarding the genera *Neisseria* and *Granulicatella* being more abundant in TG, both taxa can be regarded as health-associated. Yamashita *et al*. were able to differentiate several types of salivary microbiomes according to periodontal health and found a high abundance of *Neisseria flavescens*, *Granulicatella adiacens* and some other taxa enriched in periodontally healthy individuals. On the other hand, individuals with a high abundance of *P. histicola*, *P. melaninogenica*, several *Veillonella* and *Streptococcus* species exhibited poorer oral health^31^. Correspondingly, Sanz *et al*. infer from recent high-throughput sequencing studies that *Neisseria* and *Granulicatella* among other taxa can be regarded as genera normally associated with oral health^32^. Taken all together, we can conclude that the differential abundances of the genera *Prevotella*, *Neisseria* and *Granulicatella*, i.e. a lower abundance of *Prevotella* spp. and a higher abundance of the latter two after the long-term use of products containing stannous ions can be seen as a beneficial effect for the entirety of the oral microbiota.

In conclusion, the analysis of the salivary microbiome after a long-term use of oral hygiene products containing SnCl_2_/AmF/NaF reflects a healthier status of the microbial community. This result was also confirmed by the clinical data on the oral health status demonstrated by the biannual caries assessment by the ICDAS-II-System for both groups^33^. The clinical data on erosion showed that the treatment group yielded a better result after four years with less increment in erosion^34^. In this respect the continual use of these products as a preventive measure for tooth erosion can be recommended for patients with a high risk of erosion and can be considered as contributing to oral health in general.

## Materials and Methods

### Study design

The study was performed as an RCT in accordance with guidelines of Good Clinical Practice and conforming to the declaration of Helsinki. Ethical approval was obtained from the local ethics committee (S-566/2012; University of Heidelberg). The study was registered at the German Clinical Trials Registry Platform (DRKS00005019, registration 2013/05/27). Previous studies have shown that endurance athletes are at a higher risk of erosive tooth wear than controls^34^. For this reason, 54 participants who performed at least five hours of endurance training per week were recruited between March and October 2013 and included in the study. Inclusion criteria were: good general health, written informed consent. Exclusion criteria were: cumulative weekly training under five hours, restrictions in oral hygiene, pregnancy or lactation, participation in another clinical study within the last 30 days, use of antibiotics within the last 30 days prior to the study and each recall time point, affiliation with the dentistry department as student or staff. The full details of the study are given elsewhere^33^. Randomization of the participants into treatment group (TG) and control group (CG) was performed by block randomization (using sequentially numbered envelopes). The numbers of participants withdrawing from the study were documented in the CONSORT flow diagram (Fig. S1).

Clinical oral examinations were performed every 6 months with assessment of dental erosion (BEWE), caries index (ICDAS II) and a professional tooth cleaning at the Department of Conservative Dentistry, University Hospital Heidelberg (Heidelberg, Germany) by one blinded calibrated examiner^33,34^. For the analysis of the salivary microbiota stimulated saliva was sampled from each participant at baseline and after the three-year treatment period using sterile paraffin gum. Each participant refrained from eating and drinking, smoking and brushing their teeth for at least 2 hours before sampling.

The participants in the TG received mouth rinse containing F/Sn (AmF/NaF/SnCl_2_; 800 ppm Sn^2+^, 500 ppm F^-^; Elmex Erosion Protection Mouthrinse, CP GABA, Hamburg, Germany) and toothpaste containing F/Sn (NaF/Sn^2+^; 3500 ppm Sn^2+^, 1450 ppm F^−^; Elmex Erosion Protection Toothpaste; CP GABA, Hamburg, Germany) and were instructed to use mouthrinse 1 × 30 s per day and toothpaste twice a day for routine oral hygiene. The participants in the CG did not receive any oral hygiene products but were given instructions to use conventional fluoridated toothpaste (1500 ppm) but no products containing stannous compounds. During the follow-up visits the compliance of the participants of both groups was verified, the brands of the oral hygiene products used by the CG were retrieved and all the instructions were repeated.

### 16S rDNA Illumina MiSeq high-throughput sequencing

To analyze the salivary microbiome we applied high-throughput DNA amplicon sequencing based on the 16S rDNA on the Illumina MiSeq platform. For DNA extraction the DNA PowerSoil Kit (Qiagen, Hilden, Germany) was used according to the manufacturer’s protocol. The microbial community was analyzed using Illumina MiSeq paired-end-sequencing with two times 300 bp read length. The amplicon library was constructed using the primers 5′-CCTACGGGNGGCWGCAG-3′ and 5′-GACTACHVGGGTATCTAATCC-3′ ^35^ for the variable regions v3-v4 including the recommended adaptors for Illumina Sequencing (MiSeq Reagent Kit v3, Illumina, Eindhoven, Netherlands). Amplification, indexing, library quantification, pooling and sequencing were performed according to the Illumina MiSeq protocol for amplicon sequencing. Filtering, denoising, merging and the taxonomic classification based on the Silva database (v132^36^; was done with the Divisive Amplicon Denoising Algorithm 2 (DADA2) pipeline^37^. For comparison, taxonomic annotation was performed using Uclust (Qiime v. 1.9.1) based on a different database (Zymo Research, Irvine, USA) to achieve further taxonomic assignment on the species level.

### Mock community

As a positive control we created an internal mock community containing the following microbial species in equal proportions as measured by OD_600_: *Streptococcus sanguinis* (DSM 20068), *Streptococcus mitis* (ATCC11843), *Porphyromonas gingivalis* (W381), *Fusobacterium nucleatum* (ATCC 25586) and *Tannerella forsythia* (ATCC 43037). The DNA of this mixture was extracted using the DNA PowerSoil Kit according to the manufacturer’s protocol and was included in the Illumina MiSeq sequencing procedure.

### Statistical analysis

All statistical analyses were performed in R (V.3.6.0). As taxa abundances are not normally distributed, non-parametric tests (Wilcoxon Rank Sum Test) were used with multiple testing correction when applicable. P-values < 0.05 were regarded as statistically significant. The species richness was determined using Chao1 (richness) and alpha diversity was analyzed using Shannon Diversity (OTU-based diversity). Beta diversity was assessed by computing weighted Bray-Curtis distances to compare microbial communities based on relative abundance for each microbial community. NMDS was performed to compare beta diversity among groups. To determine features most likely to explain differences between TG and CG, we applied the linear discriminant analysis (LDA) effect size (LEfSe) method for differential abundance analysis^38^.

## Supplementary information

Supplementary Information. Tables S1-S3, Figures S1-S7

Supplementary Information 2. Table S4

Supplementary Information 3. Table S5

## Data Availability

The datasets supporting the conclusion of this article are available through GenBank (SRA accession: PRJNA577839; SUB6429482; accession numbers SRX7007447 - SRX7007521 and can be found at https://www.ncbi.nlm.nih.gov/sra/PRJNA577839 after the release date.
