# Supplementary material for: Influence of the long-term use of oral hygiene products containing stannous ions on the salivary microbiome – a randomized controlled trial
Source: Sci Rep. 2020 Jun 12;10:9546. doi: 10.1038/s41598-020-66412-z (PMC7293238; doi:10.1038/s41598-020-66412-z)

**Supplementary Information File**

**Influence of the long-term use of oral hygiene products containing stannous ions on the salivary microbiome – a randomized controlled trial**

Anderson AC^1^*, Al-Ahmad A^1^, Schlueter N^2^, Frese C^3^, Hellwig E^1^, Binder N^4^

^1^Department of Operative Dentistry and Periodontology, Faculty of Medicine, Freiburg, University of Freiburg, Germany

^2^Division for Cariology, Department of Operative Dentistry and Periodontology, Medical Center- University of Freiburg, Faculty of Medicine, University of Freiburg, Germany

^3^Department of Conservative Dentistry, Clinic for Oral, Dental and Maxillofacial Diseases, University Hospital Heidelberg, Heidelberg, Germany

^4^Institute for Prevention and Cancer Epidemiology, Faculty of Medicine, University of Freiburg; Institute of Digitization in Medicine, Faculty of Medicine, University of Freiburg, Freiburg, Germany

Address correspondence to:

Dr. Annette Carola Anderson

Department of Operative Dentistry and Periodontology,

Hugstetter Straße 55

79106 Freiburg

Germany

annette.anderson@uniklinik-freiburg.de

Supplementary Table S1:

Demographic and clinical data of the study participants (nd = not determined; t = 0 years and t = 3 years). The clinical variables regarding saliva, i.e. salivary pH and saliva flow rate were not significantly different between the two groups over the study period ([Frese et al. 2018](#_ENREF_1)).

| Study participant | Sample | group | age | | BMI [kg/m^2^] | sex  1= male  2=female | Saliva pH | | Saliva flow rate  [ml/min] | |
| --- | --- | --- | --- | --- | --- | --- | --- | --- | --- | --- |
|  |  |  | t = 0 | t = 3 | t = 0 |  | t = 0 | t = 3 | t = 0 | t = 3 |
| 1 | F01 | TG | 22 | 25 | 25.54 | 1 | 7 | 7.8 | 1.5 | 1.4 |
| 2 | F02 | TG | 33 | 36 | 21.33 | 1 | 7 | 7.4 | 2.4 | 4 |
| 3 | F04 | TG | 23 | 27 | 22.77 | 2 | 6.6 | 7.6 | 1 | 1.4 |
| 4 | F05 | TG | 57 | 60 | 19.28 | 2 | 6.2 | 6.4 | 2 | 2.8 |
| 5 | F06 | TG | 26 | 29 | 21.91 | 1 | 6.8 | 6.8 | 1.4 | 2.2 |
| 6 | F08 | TG | 45 | 48 | 23.89 | 1 | 7 | 7.2 | 2.6 | 2.6 |
| 7 | F09 | TG | 44 | 47 | 22.91 | 1 | 7.4 | 7.6 | 1.8 | 4 |
| 8 | F12 | TG | 34 | 37 | 29.84 | 1 | 6.6 | 7.6 | 1.4 | 2.2 |
| 9 | F15 | TG | 48 | 51 | 26.29 | 1 | 6.4 | 7 | 2 | 1.4 |
| 10 | F16 | TG | 30 | 33 | 24.49 | 1 | 6.6 | nd | 2.6 | nd |
| 11 | F17 | TG | 25 | 28 | 19.68 | 2 | 6.8 | 5.2 | 1.8 | 1.4 |
| 12 | F19 | TG | 48 | 51 | 23.24 | 1 | 7 | 6.6 | 3 | 2 |
| 13 | F23 | TG | 22 | 25 | 21.48 | 2 | 6.8 | 6.8 | 1.4 | 1.6 |
| 14 | F24 | TG | 35 | 38 | 22.04 | 1 | 7 | 7.6 | 3.6 | 4 |
| 15 | F25 | TG | 43 | 47 | 22.00 | 1 | 6.2 | 6.6 | 1.4 | 2 |
| 16 | F26 | TG | 38 | 41 | 22.34 | 1 | 6.8 | 7.6 | 1.8 | 2 |
| 17 | F32 | CG | 26 | 29 | 21.72 | 1 | 7 | 7.4 | 4 | 4 |
| 18 | F33 | CG | 41 | 44 | 20.88 | 1 | 5.8 | 7 | 1.2 | 2 |
| 19 | F34 | CG | 26 | 29 | 22.96 | 1 | 6.2 | 7.4 | 2 | 1.2 |
| 20 | F35 | CG | 31 | 34 | 23.99 | 1 | 7.6 | 7.6 | 1.6 | 1 |
| 21 | F37 | CG | 44 | 47 | 22.06 | 1 | 6.6 | 7 | 1.8 | 2 |
| 22 | F38 | CG | 24 | 27 | 20.98 | 1 | 6.8 | 7.4 | 2 | 2.6 |
| 23 | F39 | CG | 36 | 39 | 22.40 | 1 | 6.8 | 6.4 | 2.2 | 1.6 |
| 24 | F40 | CG | 43 | 47 | 25.77 | 1 | 6.6 | 7 | 1.2 | 3 |
| 25 | F41 | CG | 52 | 55 | 19.10 | 2 | 7.2 | 7.8 | 3 | 4 |
| 26 | F42 | CG | 36 | 39 | 22.22 | 1 | 6.6 | 6.6 | 0.7 | 0.7 |
| 27 | F43 | CG | 39 | 42 | 23.53 | 1 | 6.6 | 6.2 | 1.4 | 2 |
| 28 | F44 | CG | 22 | 25 | 20.52 | 1 | 7 | 7 | 1.8 | 3 |
| 29 | F46 | CG | 32 | 35 | 21.72 | 1 | 6.8 | 7 | 1.2 | 2 |
| 30 | F47 | CG | 27 | 30 | 24.00 | 2 | 6.6 | 7.2 | 1.6 | 1.6 |
| 31 | F48 | CG | 50 | 53 | 24.26 | 1 | 6.6 | 6.2 | 1 | 1.2 |
| 32 | F49 | CG | 35 | 38 | 23.77 | 1 | 6.8 | 7.4 | 2.6 | 2.8 |
| 33 | F51 | CG | 42 | 45 | 19.49 | 2 | 6.6 | nd | 1.2 | nd |
| 34 | F52 | CG | 48 | 51 | 22.40 | 1 | 6.4 | 6.4 | 2.6 | 2.2 |
| 35 | F53 | CG | 37 | 40 | 23.91 | 1 | 7.2 | 7.4 | 1.6 | 2.2 |
| 36 | F54 | CG | 26 | 29 | 24.33 | 1 | 6.6 | 6.2 | 1.4 | 2.4 |
| 37 | F55 | CG | 46 | 49 | 26.30 | 1 | 6.6 | nd | 1.6 | 1.4 |
| 38 | F56 | CG | 41 | 44 | 29.94 | 1 | 6.8 | nd | 3.4 | nd |

Supplementary Table S2

Alpha-Diversity indices (zymoresearch data) for TG and CG (t = 3 years)

| Study participant | Sample nr | Group | Shannon | Simpson | Simpson effective | Simpson reciprocal |
| --- | --- | --- | --- | --- | --- | --- |
| 1 | F01 | TG | 4.99 | 0.94 | 0.16 | 16.77 |
| 2 | F02 | TG | 4.85 | 0.94 | 0.27 | 17.23 |
| 3 | F04 | TG | 4.90 | 0.95 | 0.24 | 18.81 |
| 4 | F05 | TG | 5.12 | 0.95 | 0.24 | 20.83 |
| 5 | F06 | TG | 5.09 | 0.96 | 0.34 | 23.29 |
| 6 | F08 | TG | 4.82 | 0.94 | 0.25 | 17.66 |
| 7 | F09 | TG | 5.45 | 0.96 | 0.23 | 24.85 |
| 8 | F12 | TG | 5.10 | 0.94 | 0.19 | 17.16 |
| 9 | F15 | TG | 5.00 | 0.95 | 0.27 | 20.09 |
| 10 | F16 | TG | 5.03 | 0.95 | 0.25 | 20.67 |
| 11 | F17 | TG | 5.17 | 0.95 | 0.25 | 21.31 |
| 12 | F19 | TG | 5.53 | 0.96 | 0.18 | 24.71 |
| 13 | F23 | TG | 5.20 | 0.95 | 0.23 | 18.56 |
| 14 | F24 | TG | 5.77 | 0.97 | 0.30 | 34.86 |
| 15 | F25 | TG | 5.32 | 0.96 | 0.39 | 27.38 |
| 16 | F26 | TG | 5.15 | 0.94 | 0.13 | 15.98 |
| 17 | F32 | CG | 5.86 | 0.96 | 0.14 | 22.73 |
| 18 | F33 | CG | 5.47 | 0.96 | 0.20 | 24.78 |
| 19 | F34 | CG | 5.03 | 0.95 | 0.29 | 19.89 |
| 20 | F35 | CG | 5.52 | 0.96 | 0.28 | 26.49 |
| 21 | F37 | CG | 5.36 | 0.95 | 0.19 | 21.52 |
| 22 | F38 | CG | 5.30 | 0.95 | 0.25 | 21.75 |
| 23 | F39 | CG | 5.42 | 0.96 | 0.26 | 26.25 |
| 24 | F40 | CG | 5.47 | 0.96 | 0.22 | 24.18 |
| 25 | F41 | CG | 5.37 | 0.94 | 0.15 | 17.08 |
| 26 | F42 | CG | 5.13 | 0.92 | 0.10 | 12.93 |
| 27 | F43 | CG | 5.30 | 0.96 | 0.32 | 26.50 |
| 28 | F44 | CG | 5.50 | 0.96 | 0.16 | 22.81 |
| 29 | F46 | CG | 5.18 | 0.96 | 0.32 | 22.85 |
| 30 | F47 | CG | 5.26 | 0.96 | 0.29 | 26.55 |
| 31 | F48 | CG | 5.69 | 0.96 | 0.16 | 26.84 |
| 32 | F49 | CG | 4.91 | 0.94 | 0.19 | 16.70 |
| 33 | F51 | CG | 4.99 | 0.95 | 0.22 | 18.23 |
| 34 | F52 | CG | 5.27 | 0.96 | 0.28 | 23.64 |
| 35 | F53 | CG | 5.57 | 0.96 | 0.26 | 27.33 |
| 36 | F54 | CG | 5.21 | 0.96 | 0.30 | 22.54 |
| 37 | F55 | CG | 5.56 | 0.95 | 0.14 | 20.42 |
| 38 | F56 | CG | 5.67 | 0.97 | 0.34 | 33.99 |

Supplementary Table S3

Percentage of sequence reads of different taxa in the mock community samples analyzed by Illumina MiSeq. Bold: Taxa included in mock community

| Genus | Sample 1 | Sample 2 |
| --- | --- | --- |
| ***Streptococcus*** | **26.15** | **30.58** |
| ***Porphyromonas*** | **33.55** | **31.82** |
| ***Tannerella*** | **3.06** | **4.08** |
| ***Fusobacterium*** | **37.13** | **33.39** |
| *Faecalibacterium* | 0.01 | 0.01 |
| *Prevotella* | 0.03 | 0.02 |
| *Haemophilus* | 0.01 | n.d. |
| *Rothia* | 0.06 | n.d. |
| *Bacteroides* | n.d. | 0.04 |
| *Rhodospirillales* | n.d. | 0.01 |
| *Oribacterium* | n.d. | 0.01 |
| *Ruminococcus* | n.d. | 0.01 |
| *Veillonella* | n.d. | 0.02 |
| *Alloprevotella* | n.d. | 0.01 |
| *Collinsella* | n.d. | 0.01 |

References

Frese C, Wohlrab T, Sheng L, Kieser M, Krisam J, Frese F, Wolff D. 2018. Clinical management and prevention of dental caries in athletes: A four-year randomized controlled clinical trial. Scientific reports. 8(1):16991.

Supplementary Figure S1

CONSORT flow diagram showing the adverse events as well as the reasons for withdrawal. Six control subjects missed one follow-up appointment each, but continued with the study and participated in the remaining follow-up examinations.


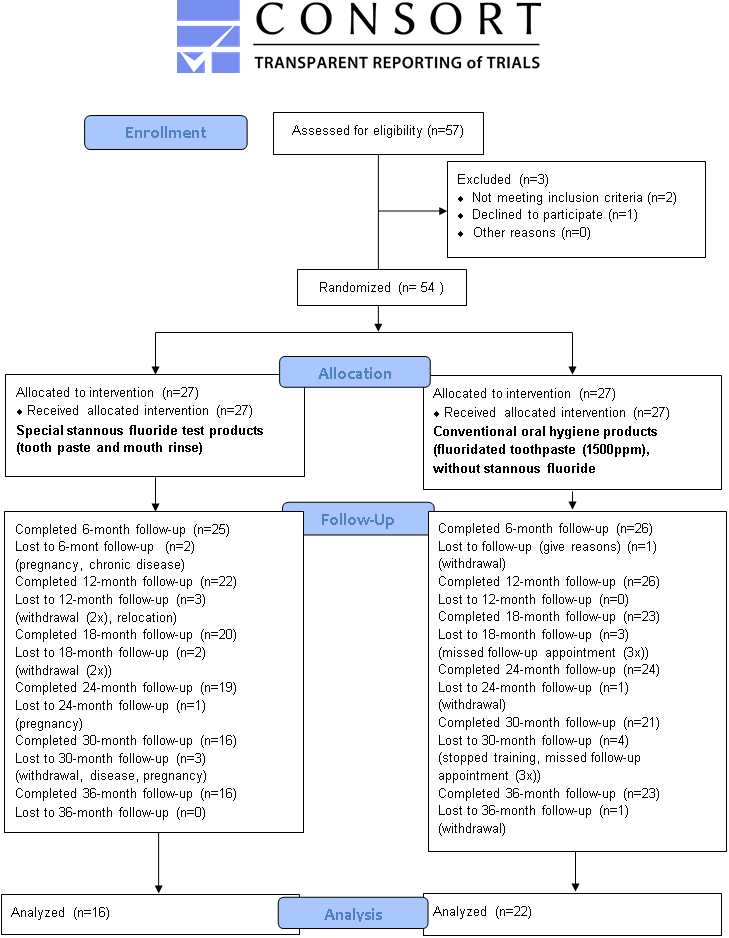


Supplementary Figure S2

Relative abundances of the bacterial phyla found in saliva in TG and CG are depicted for each study participant (F01 – F56) (t = 3 years).


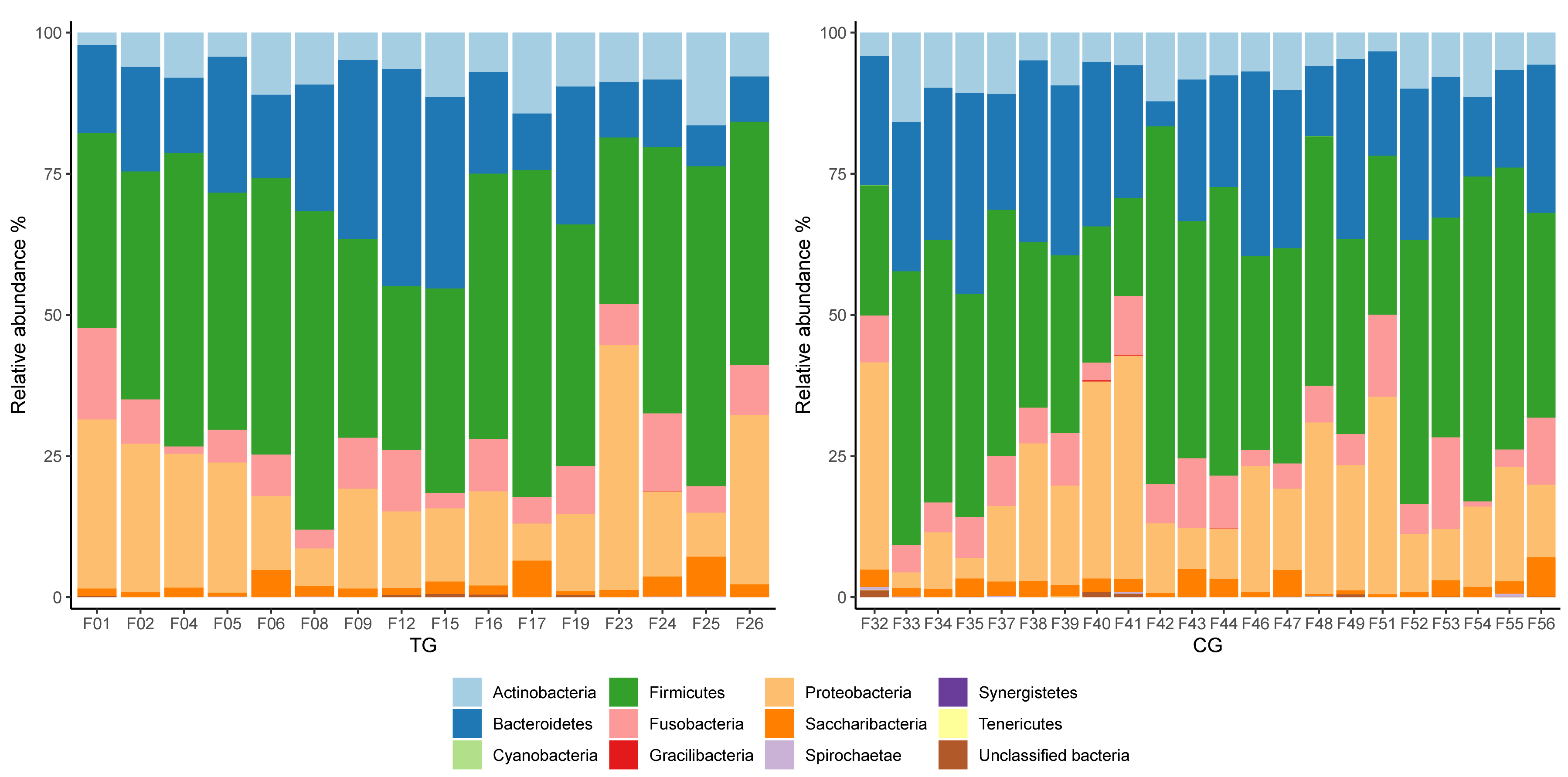


Supplementary Figure S3

Relative abundances of the bacterial phyla (>1% abundance) found in saliva in TG and CG at baseline (t = 0 years).


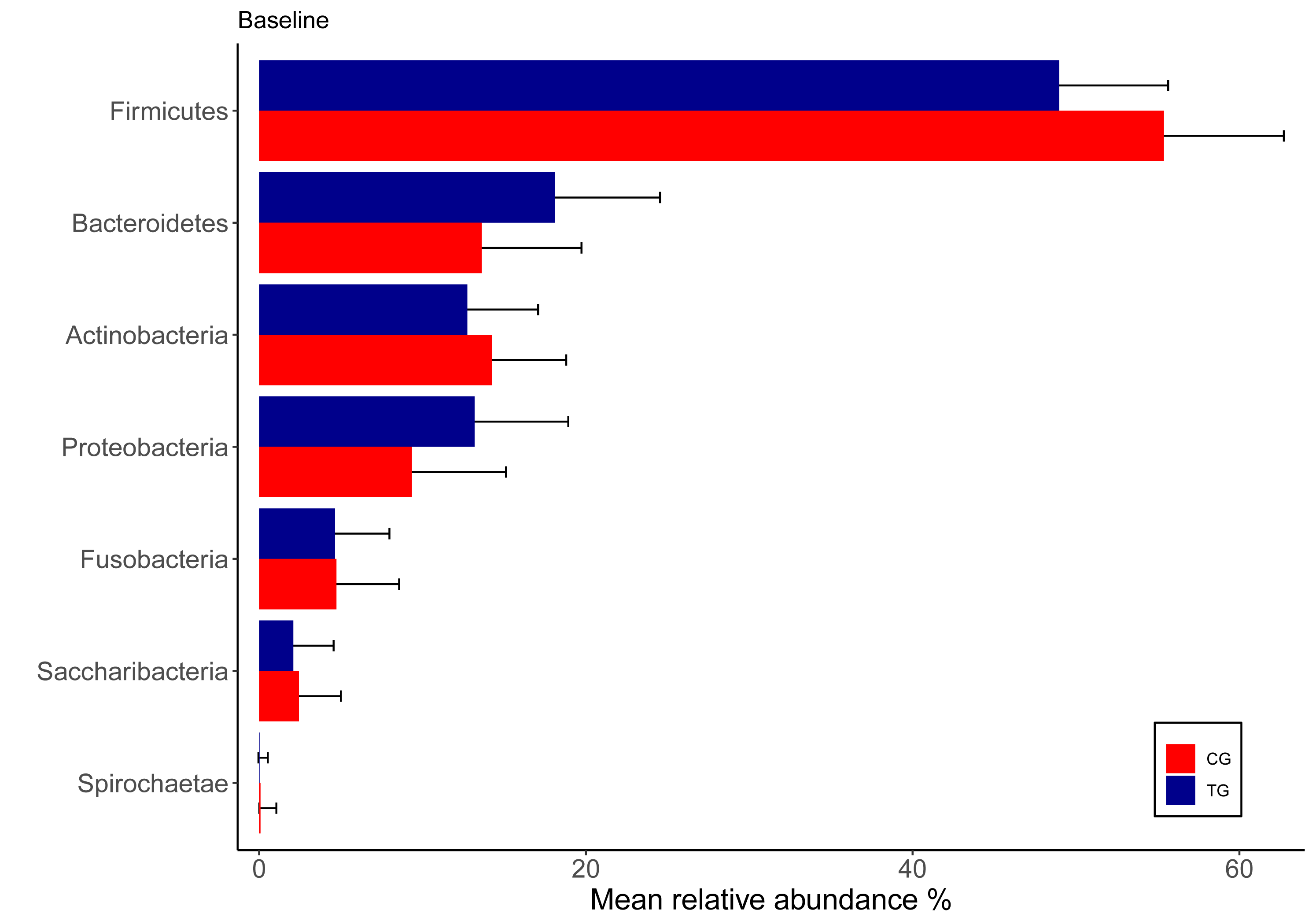


Supplementary Figure S4

Relative abundances of the bacterial phyla found in saliva in TG and CG at baseline (t = 0 years) are depicted for each study participant (S01 – S56).


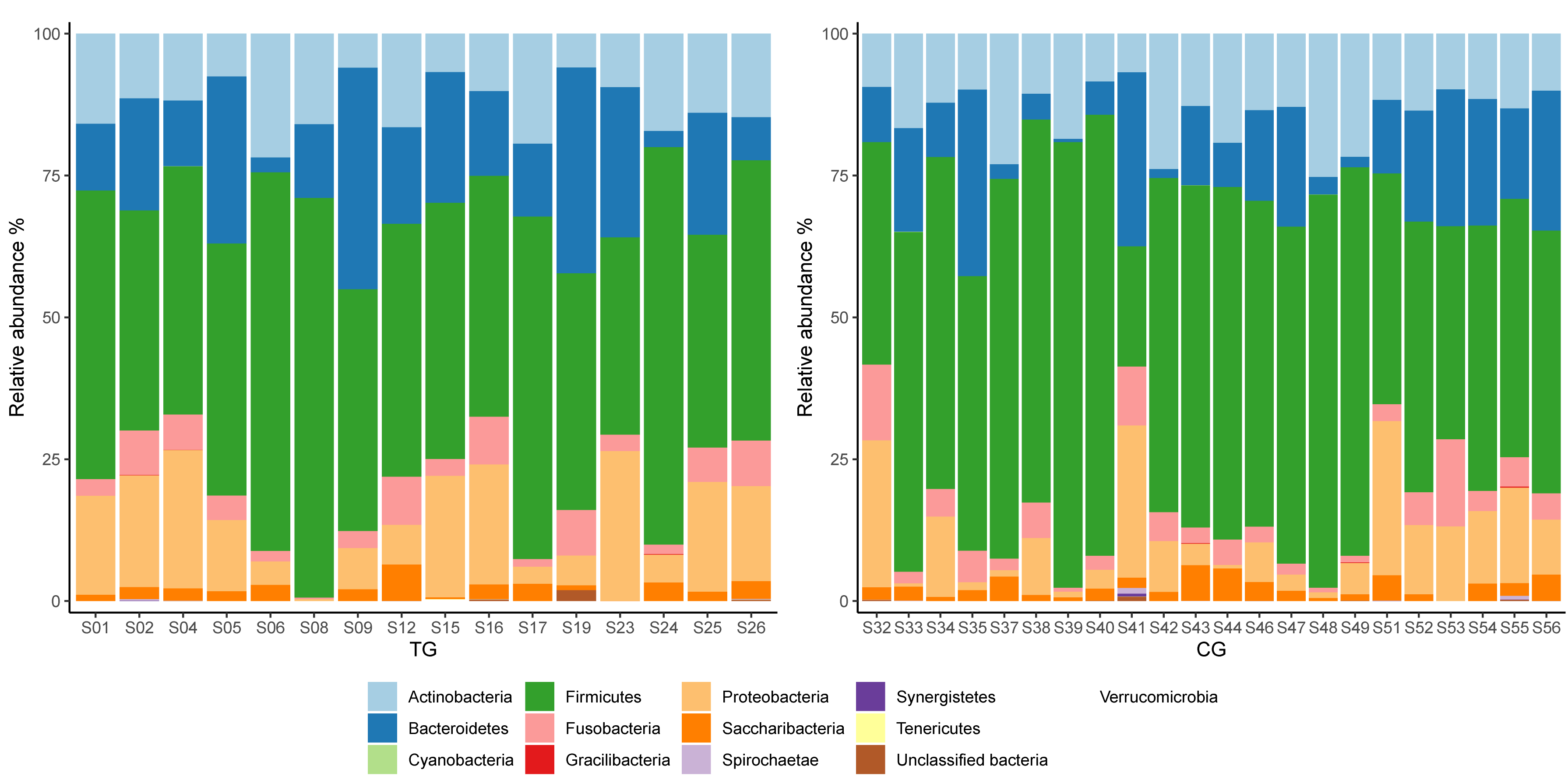


Supplementary Figure S5

Relative abundances of the bacterial genera (>1% abundance) found in saliva in TG and CG at baseline (t = 0 years).


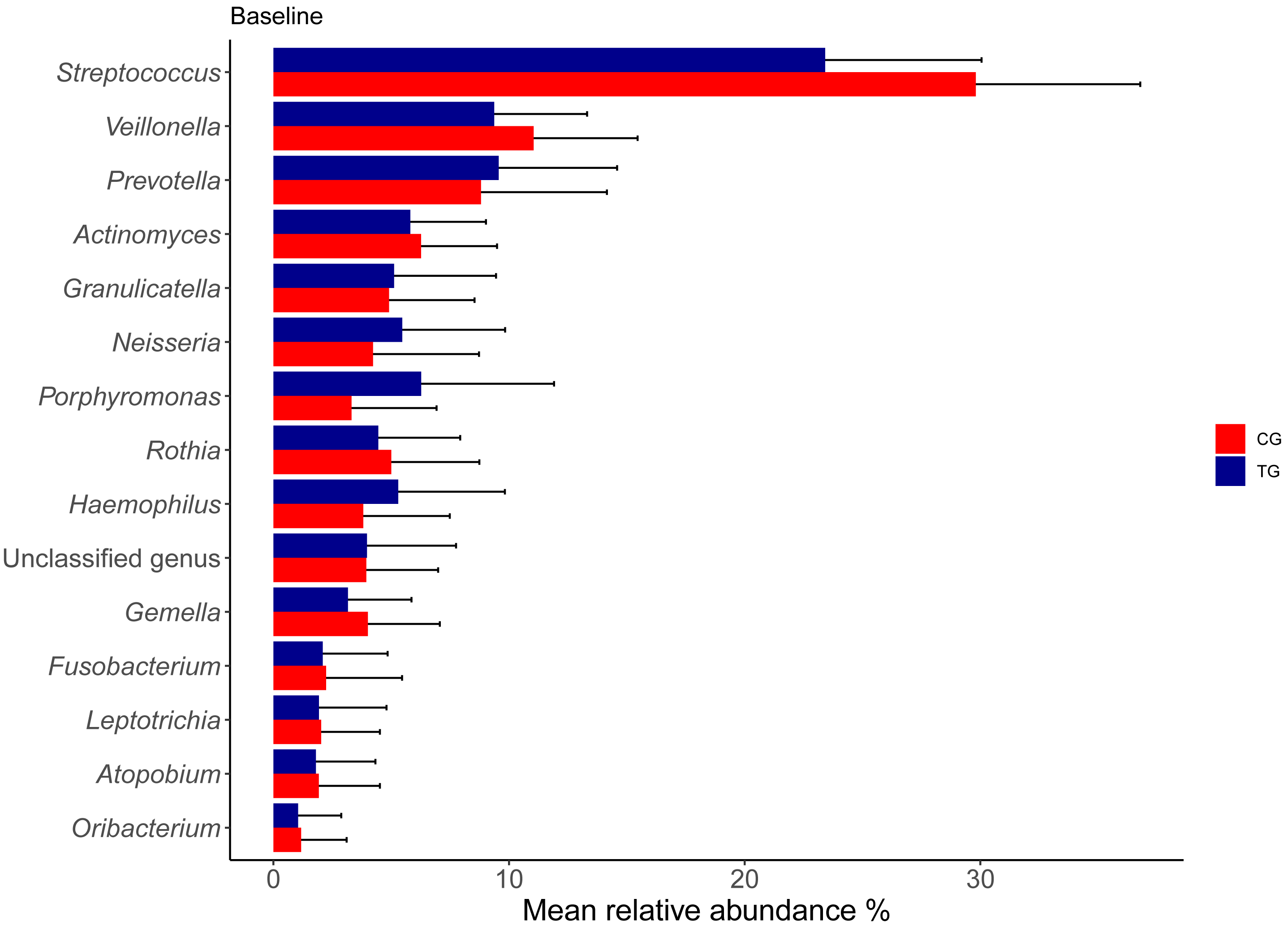


Supplementary Figure S6

Relative abundances of the different *Prevotella* species found in saliva at follow-up (t=3) in TG and CG are depicted for each study participant (F01 – F56).


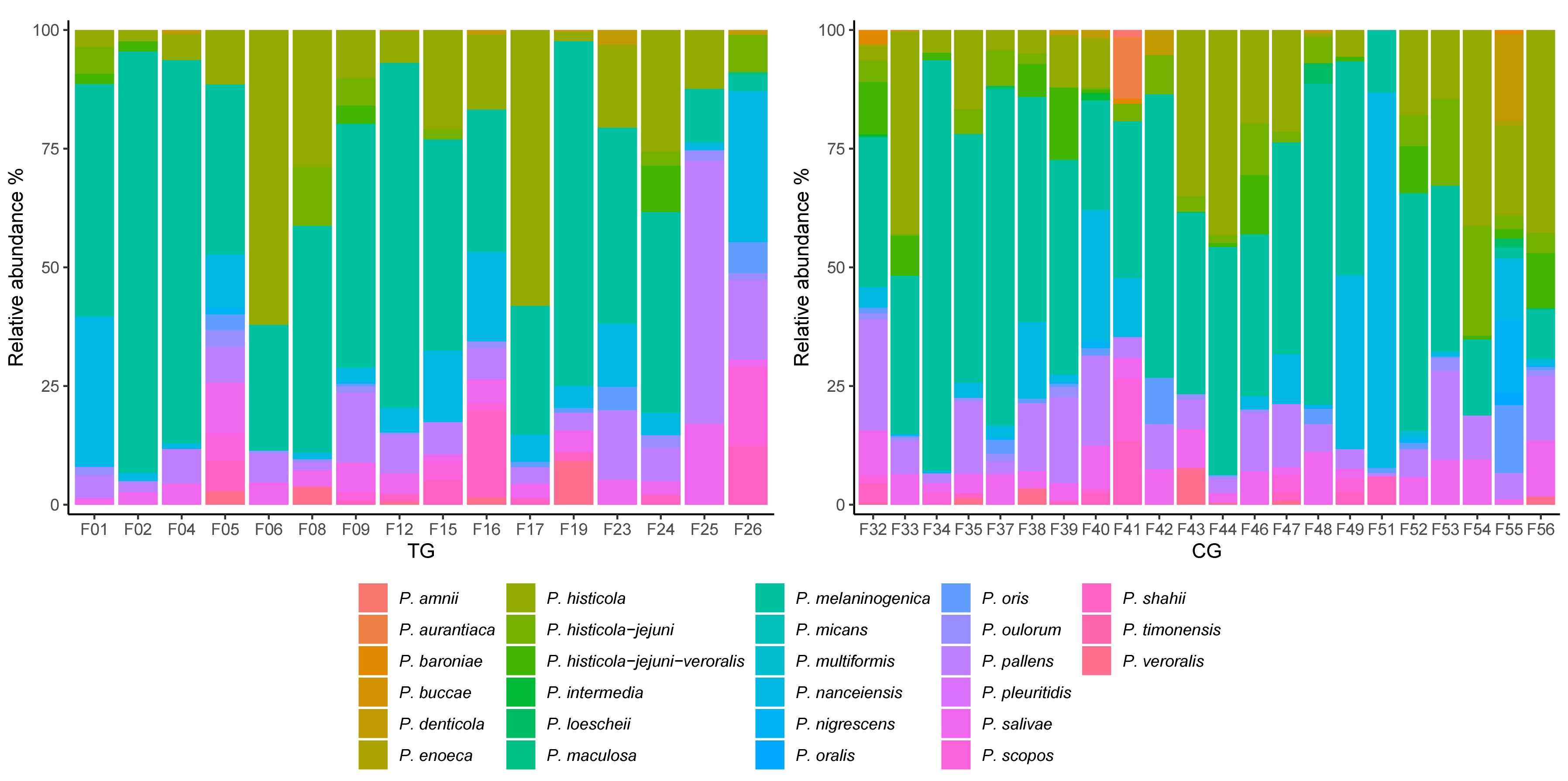


Supplementary Figure S7

Relative abundances of the genera *Prevotella*, *Streptococcus* and *Porphyromonas* in TG and CG (Control group) at baseline (t = 0 years); NS. = not significant.


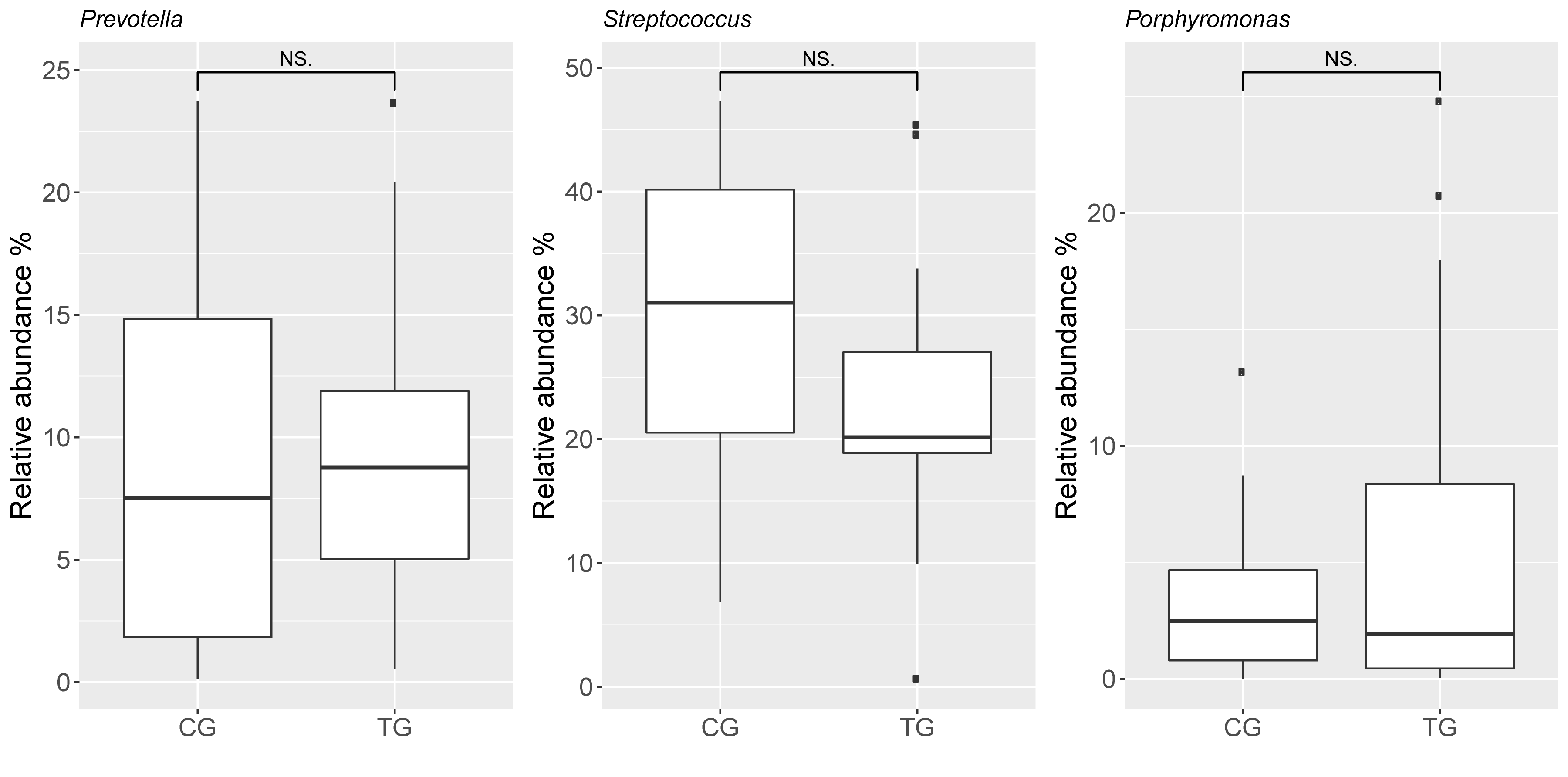

Supplement: Supplementary file 1 — Supplementary Information. Tables S1-S3, Figures S1-S7 [file 41598_2020_66412_MOESM1_ESM.docx]
